# Pelvic mass associated with raised CA 125 for benign condition: a case report

**DOI:** 10.1186/1477-7819-8-28

**Published:** 2010-04-16

**Authors:** Viren Asher, Robert Hammond, Tim J Duncan

**Affiliations:** 1 Department of Obstetrics and Gynaecology, Royal Derby Hospital, Uttoxeter road, Derby DE22 3NE, UK; 2 Department of Gynaecological oncology, Nottingham City Hospital, Hucknall road, Nottingham NG5 1PB, UK; 3 Department of Gynaecological Oncology, Norfolk and Norwich University Hospitals NHS Trust, Conley Lane, Norwich, Norfolk, UK

## Abstract

**Background:**

Raised CA 125 with associated pelvic mass is highly suggestive of ovarian malignancy, but there are various other benign conditions that can be associated with pelvic mass and a raised CA 125.

**Case presentation:**

We present a case of 19 year old, Caucasian British woman who presented initially with sudden onset right sided iliac fossa pain and on imaging was found to have 9.8 × 4.5 cm complex cystic mass in right adnexa with a raised CA 125 of 657, which was initially thought to be highly suspicious of cancer but was subsequently found to be due to pelvic inflammatory disease on histology.

**Conclusion:**

This case highlights the fact that though a pelvic mass with raised CA 125 is highly suggestive of malignancy, pelvic inflammatory disease should always be considered as a differential diagnosis especially in a young patient and a thorough sexual history and screening for pelvic infection should always be carried out in these patients.

## Background

The detection of pelvic mass with an associated elevated CA 125 is highly suspicious of ovarian cancer, but there are various benign conditions which mimic the above findings, especially in premenopausal women.

## Case presentation

A 19 year old nulliparous, British Caucasian woman was admitted with a sudden onset of right iliac fossa pain. Urine pregnancy test was negative. This pain was sharp and stabbing in nature with no radiation. There was no associated vomiting or fever. She denied any urinary urgency, frequency or dysuria and her bowels were normal. On examination there was minimal guarding and no rebound tenderness. No distension was seen and bowel sounds were heard. Transvaginal pelvic ultrasound demonstrated two small simple cysts within the right ovary. She was managed conservatively with analgesics only and the pain resolved within 24 hours. Following this acute episode she developed intermittent pelvic pain. Her subsequent scan showed 9.8 × 4.5 cm complex cystic mass in right adnexa with features suggestive of a dermoid cyst with no colour flow on Doppler examination. Interestingly her CA 125 was markedly elevated at 657; CEA, ∝ FP, HCG, white cell count (WCC) and CRP were all within normal limits. Her periods were regular and she was using condoms for contraception. She was in a new relationship and they had been together for the last 4 months.

Past medical history included well controlled asthma, a negative laparotomy at the age of seven for abdominal pain but no previous pelvic infections. Pelvic examination revealed a normal size uterus with a right adnexal mass which appeared fixed to the pelvic side wall.

A subsequent CT scan one week later suggested a right adnexal dermoid cyst 5.4 × 4.8 cm with abnormal soft tissue 3.0 × 2.6 cm deep to right rectus muscle and abnormal irregular soft tissue along pelvic side wall extending from left common iliac bifurcation to left adnexa and an enlarged 10 mm precaval lymph node was also seen. These features were thought to be highly suspicious of malignancy during the case review at the Gynaecology oncology Multidisciplinary Team (MDT) meeting.

A further CA 125 level was measured pre-operatively and had fallen to 342. A provisional diagnosis of either pelvic inflammatory disease, endometriotic cyst or an ovarian malignancy was made. She underwent a midline laparotomy that revealed right ovarian cyst (7 × 6 × 6 cm), with associated hydrosalpinx. The tubo-ovarian mass was adherent to the terminal ileum, caecum and omentum with appendix buried in the mass. The left ovary was normal, although there was evidence of a left pyosalpinx, which was drained to conserve left tube. A right salpingo-opherectomy, appendicectomy and omental biopsy was performed and an intraoperative swab from the mass was sent for culture and sensitivity. She had high vaginal, endocervical and Chlamydia swabs postoperatively and her recovery was uneventful.

Histology revealed a benign cystic ovarian teratoma, the fallopian tube showed acute on chronic salpingitis and the appendix was normal. The post operative endocervical swab was positive for Chlamydia. Both the partners were then subsequently referred to Genito- urinary clinic for ongoing treatment and contact tracing.

## Discussion

Ovarian cancer is the second most common female gynaecological cancer in the UK with 6,806 cases detected in 2005 and the lifetime risk of developing the disease is 1 in 48 [1]. The majority of these are detected in advanced stages contributing to the poor prognosis of this disease[2]. The incidence of ovarian cancer is low in young women and epithelial ovarian cancers are not known to occur before menarche, and most of them (though rare) are germ cell tumour, juvenile granulosa cell tumour and serous borderline tumours. Age specific incidence is 40/100,000 by the age of 50 and rises to 50 per 100,000 women by the age of 65 yrs[3].

For early detection of ovarian cancer various tumour markers have been studied and CA 125 has been proposed by Bast et al[4] as a relatively specific marker for ovarian cancer. The CA 125 molecule is a 200-kDa glycoprotien and was initially identified on the surface of the ovarian carcinoma cell line OVCA433[5]. CA 125 is widely distributed on the surface of both healthy and malignant cells of mesothelial origin, including pleural, pericardial, peritoneal and endometrial cells, as well as in normal genital tract and amniotic membrane. Interestingly the molecule is not present on the surface of normal ovarian cells, but is present in 80% of malignant ovarian tissue of non mucinous origin[3]. The value of CA 125 varies between laboratories depending on the type of assay used but levels < 35 kIU/L are considered to be normal[6].

In view of wide distribution of CA 125 expression, serum CA 125 levels can be raised in various benign and inflammatory conditions such as menstruation, pregnancy, endometriosis, pelvic inflammatory disease and non- gynaecological conditions including various liver and pulmonary diseases.

Differentiating benign from early malignant ovarian disease is important and provides a diagnostic challenge. The combination of pelvic mass and elevated level CA 125 arouses suspicion of a gynaecological malignancy, but other conditions should always be considered in the differential diagnosis, especially in a pre menopausal female. Malkasion[7] studied 59 patients with histologically proven benign ovarian cysts. Out of these patients 17 had elevated concentrations of CA 125 (12 > 35 units/ml, 4 > 65 units/ml and 1 > 2000 units/ml). In another study by Dixia[8] using 153 patients with benign pelvic masses, 10 patients had CA 125 concentrations >188 units/ml and one patient had a value of more that 400 units/ml. Nolen et al screened 65 biomarkers in patients with adnexal masses and more than half of the biomarkers differed significantly between benign and malignant masses. CA 125 and HE4 in combination provided the highest discrimination between benign and malignant cases[9]. These studies demonstrate that using CA 125 in isolation has a limited value in differentiating benign from malignant pelvic masses. The patient characteristics and radiological information provides crucial additional information on which to base a diagnosis.

Pelvic ultrasound in conjunction with CA 125 represents the most frequently utilised investigations for patients with adnexal masses. CT scan has limited value in the initial assessment of adnexal masses due to poor soft tissue discrimination and with disadvantages for irradiation[10], but can help to assess the extent of disease in the upper abdomen prior to primary cytoreduction and following chemotherapy to detect resistant disease or recurrence[11]. The CT scan in the current case was misleading, with irregular pelvic side wall soft tissue and pre-caval lymph node assumed to be malignant most likely representing inflammation from the Chlamydia infection. MRI has also been suggested to be helpful in detection of organ of origin for pelvic masses. MRI has a sensitivity of 96% while it was only 77% for Ultrasound and 87% for CT for detection of pelvic masses. MRI has been shown to correctly identify organ of origin in 94% compared to only 66% of Ultrasound[12]. Review of literature from 1990 to 2006 which included 143 studies showed that Ultrasound findings were similar to CT and MRI in differentiation of benign from malignant ovarian masses[13]. Currently newer imaging in the form of Positron emission tomography (PET) and CT can be used to judge the extent of the disease and also differentiate between malignant and benign masses [14]. As it is evident from above studies all the modalities are complimentary to each other with ultrasound remaining the first diagnostic modality as it is cheap and widely available in all units. Further assessment of the spread of disease can either be made by CT or MRI and PET scanning where facilities exist.

As the CA 125 molecule is identified in normal peritoneal and fallopian tubes, it is not surprising that inflammation of these tissues can result in an increased concentration of serum CA 125. Ruibal et al[15] found that nine of twelve women with suspected peritonitis had CA 125 concentrations of > 65 units/ml with two patients having value > 500 units/ml. A more definitive study examined CA 125 values in 30 patients with pelvic inflammatory disease associated with fever who had a good response to antibiotic therapy. CA 125 > 100 units/ml was seen in 5 patients (17%) and the highest value was 550 units/ml[16]. This increased serum concentration of CA 125 can be explained by the local expression by the inflamed tissue. Another study of 33 patients with pelvic inflammatory disease showed that 32 patients had increased concentrations of CA 125 with values between 100 and 1300 units/ml[17].

In the current case the key finding of a reduction in CA 125 between the serial measurements suggested that the elevation witnessed may be of benign origin. This is reflected in the well documented exponential rise in CA 125 levels described in ovarian malignancy[18].

## Conclusion

The presence of a pelvic mass with a raised CA 125 of 657 units/ml, lymphadenopathy and other associated suspicious features on CT scan suggested an ovarian malignancy. A subsequent fall of CA 125 to 342 units/ml pointed to an inflammatory or benign condition. The mass on laparotomy was found to be associated with pelvic inflammatory disease. Raised CA 125 levels can be misleading, as illustrated in this case, a differential diagnosis of pelvic inflammatory condition should always be considered in young patients. These patients when present with adnexal mass, it is important to elicit a detailed sexual history with specific emphasis on previous pelvic inflammatory disease. Screening women for pelvic infection using a high vaginal swab, endocervical swab and Chlamydia swab, when presenting with pelvic pain is essential, even if a likely cause such as a pelvic mass is already detected.

## Consent

Written informed consent has been obtained from the patient for publication of this case report.

## Competing interests

The authors declare that they have no competing interests.

## Authors' contributions

VH was involved in pre and post operative care of the patient and wrote the manuscript. RH and TJD performed the surgery and helped in correction of the manuscript. All authors have read, approved and contributed towards the manuscript.
